# Safe and effective removal of cyanoacrylate vascular access catheter securement adhesive in neonates

**DOI:** 10.3389/fped.2023.1237648

**Published:** 2023-08-25

**Authors:** Kevin Hugill, Matheus F. P. T. van Rens, Angela Alderman, Lori Kaczmarek, Carolyn Lund, Amy Paradis

**Affiliations:** ^1^ Nursing and Midwifery Education, Hamad Medical Corporation, Doha, Qatar; ^2^ Neonatology Department, Radboud University, Nijmegen, Netherlands; ^3^ NICU, Carilion Children’s Hospital, Roanoke, VA, United State; ^4^ Adhezion Biomedical, LLC, Wyomissing, PA, United State; ^5^ NICU, UCSF Benioff Children's Hospital, Oakland, CA, United State; ^6^ School of Nursing, University of California, San Francisco, CA, United State; ^7^ NICU, CNS Doctors Medical Center, Modesto, CA, United State

**Keywords:** neonate, complications, neonatal intensive care unit (NICU), vascular catheters, tissue adhesives cyanoacrylate, medical adhesive-related skin injury (MARSI)

## Abstract

Neonatal vascular access continues to pose challenges. Recent times have seen considerable innovations in practice and the design and manufacture of materials used to provide infusion-based therapies with the intent of reducing the incidence and severity of vascular access-related complications. However, despite these efforts, vascular access-related complication rates remain high in this patient group and research evidence remains incomplete. In neonates, a medical-grade formulation of cyanoacrylate adhesive is widely used to secure percutaneously inserted central venous catheters and is beginning to establish a role in supporting the effective securement of other devices, such as umbilical and peripheral intravenous catheters. This Perspective article considers issues specific to the removal of cyanoacrylate used to secure vascular access devices from neonatal skin before its bonding releases due to natural skin exfoliation processes. The aim of this information is to ensure the safe and effective removal of octyl-cyanoacrylate adhesive-secured vascular access catheters from neonatal skin and stimulate professional discussion.

## Introduction

Over recent years, evidence-based practice innovations, such as care bundles, and intravenous (IV) team approaches have become firmly embedded in neonatal vascular access (VA) practice (1–4). These developments have taken place alongside technological improvements in the design and manufacture of VA devices and catheters for neonatal patient populations, with the intent of reducing VA-related complications (5, 6). Despite these advances, the incidence of VA-related complications remains unacceptably high, and dwell times for VA catheters, particularly peripheral IV catheters (PIVC) among this patient group, are generally considered to be suboptimal (6, 7).

Improving the securement of vascular catheters is a challenge for practitioners. Inadequate securement can lead to therapy failure and the unplanned removal of the catheter (1, 2). This can lead to infants being exposed to repeated painful VA procedures and critical delays in therapy. Adverse experiences like these have been associated with poorer outcomes for infants and their families (1, 6, 8–11).

## Discussion

Medical grade cyanoacrylate tissue adhesives (TAs) are available in various chemical formulations, and these are typically based on octyl, butyl, or isobutyl esters. TAs form strong bonds between contact surfaces when exposed to ambient moisture. Individual products differ in their adhesive characteristics, breaking strength, and flexibility and are marketed under a range of brand names for specific clinical uses. TAs have established a role in traumatic and surgical wound closure (12, 13), and in securing vascular catheters (1, 2, 14). TAs formulated for use in VA are referred to as catheter securement cyanoacrylate adhesives (CSCAs) to differentiate them from other TAs (15). There are few absolute contraindications to CSCA use but some medical device composition incompatibilities, allergic sensitivity to components, and certain skin conditions are detailed in product indications for use (IFU) (1, 12–20).

### Securing vascular access devices in neonates

Historically, neonatal clinicians have relied upon medical adhesive tapes and surgical sutures for catheter securement. Some types of tape pose a significant risk of medical adhesive-related tissue injury (MARSI) in this vulnerable patient population (16–18, 21–22) due to the interaction of intrinsic and extrinsic factors, including anatomical and physiological skin barrier immaturity, skin maturation processes, frequent invasive procedures, and therapeutic medical device use (16, 22).

Recently, engineered catheter securement devices and a CSCA have entered practice (1, 2, 15, 23–29). Initially, these approaches were limited to central vascular catheter (CVC) securement but are now recommended as an adjunct for peripheral intravenous catheter (PIVC) securement (1, 19, 28). Currently, only one product, marketed as SecurePortIV® (Adhezion Biomedical, Wyomissing PA, USA), has Food and Drug Administration (FDA) and Certificate European (CE) approval for use in VA across all patient age groups and catheter types (1, 2, 20). There is an emerging body of empirical evidence from neonatal studies that this CSCA formulation is safe to use and can achieve the benefits reported in studies with other patient age groups (27–29).

**Figure 1:**
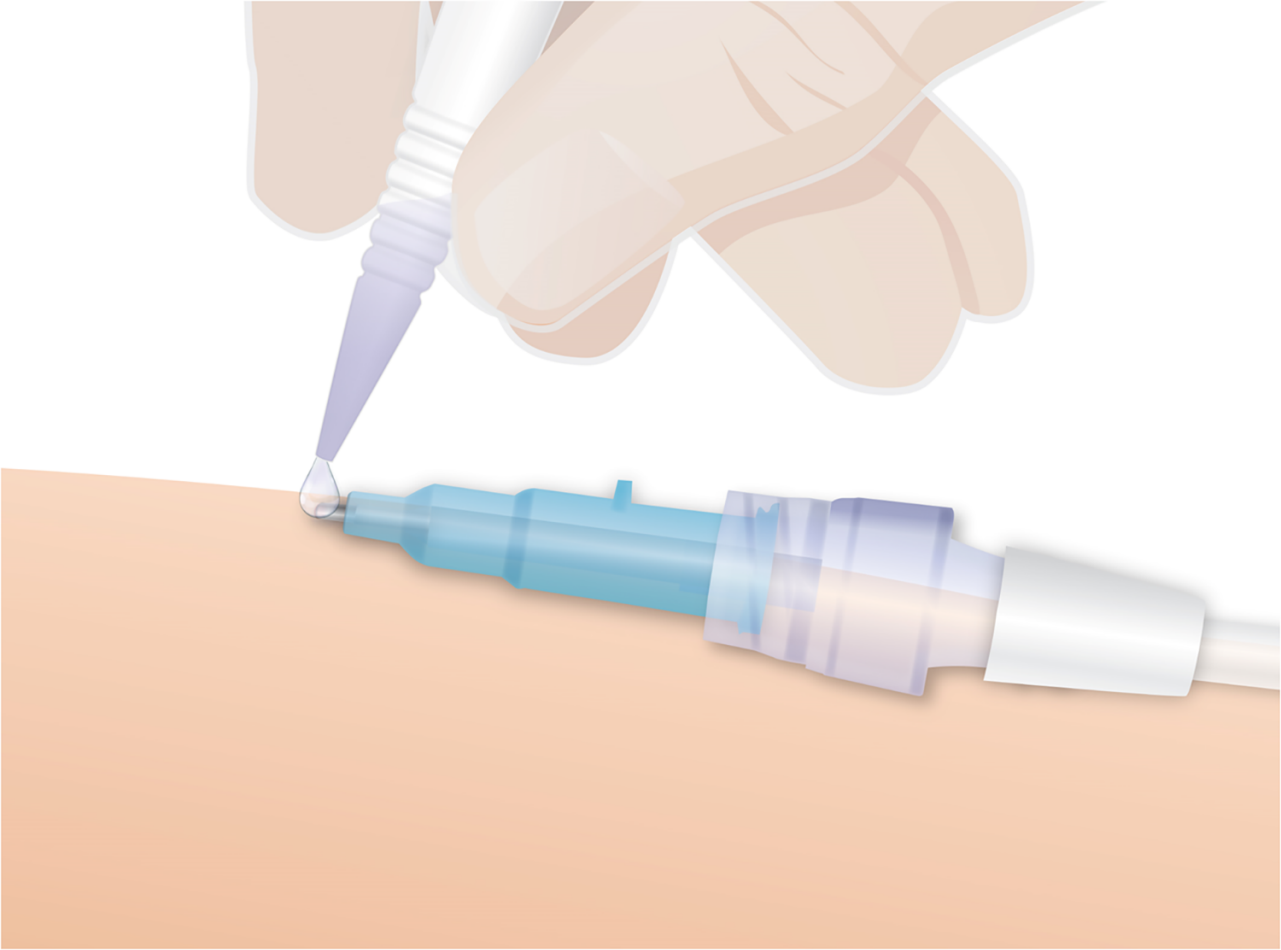
Applying CSCA. Image courtesy of Adhezion Biomedical, LLC, Wyomissing, PA, USA, used with permission from Adhezion Biomedical.

The CSCA formulation used in VA has an established safety record, but MARSI remains a risk, particularly if the product is over-applied (16, 17, 21, 24). A characteristic of CSCA is that the adhesive bond forms more efficiently when thin layers are applied, as thicker layers can lead to less effective catheter securement. Consequently, it is essential to avoid overapplication; normally only 1–2 drops are required to achieve effective securement (Figure 1) (20).

Applied correctly, CSCAs are painless, aid insertion site closure, and promote hemostasis. They provide effective securement, reduce catheter migration, the risk of accidental removal, and decrease the need for frequent dressing changes (15, 23). Furthermore, CSCAs inhibit bacterial and fungal growth, with evidence supporting reductions in the risk of acquiring catheter-associated infection (1, 2, 5, 15, 24, 27–30). Standards and guidelines for catheter securement recommend that CSCA, used alone or combined with tapes, other securement devices, and the primary transparent dressing can reduce VA-related complications (1, 2, 19, 26, 31).

In practice, there are three common situations when early (before the natural separation of the adhesive from exfoliated skin cells) CSCA removal may be required. First, and most common, is to facilitate the repositioning of the CVC tip shortly after insertion of the catheter. Second, is elective catheter removal following the completion of therapy. Finally, the removal of a catheter after infusion-related complications, such as leakage, occlusion, peripheral IV infiltration/extravasation (PIVIE), or catheter dislodgment, is another unplanned removal situation.

### Recommendations for best practice

#### General considerations

To ensure best practice overall, rather than in isolated patches, it is essential that all VA-related procedures are carried out with due attention to the wider context of patient care. Before beginning, inform the infant's parents about the procedure and the reason for doing it; this will provide reassurance, help to build more effective therapeutic relationships, and could potentially provide an opportunity for greater parental involvement (9–11, 32). To ensure patient safety and avoid the risk of harm, verify the correct patient, correct device and site, and rationale for catheter removal (33). During catheter removal, adhere to routine infection control and personal protective equipment (PPE) practices to ensure personal and patient safety (e.g., 34–37).

A key element of practice is to ensure that neurodevelopmentally appropriate practices are consistently and universally implemented (10, 11). For example, practitioners should consider individual intrinsic patient characteristics and extrinsic risks before attempting CSCA removal while ensuring supportive thermoregulation throughout. Additionally, individual behavioral cues, assessment of sleep/wake patterns, the relationship of this activity to other care activities or painful procedures, and the degree of clinical urgency should be considered when determining when it is best to remove a catheter (10).

Ensuring that comfort measures are in place throughout is essential for minimizing pain experiences. Recall the reason for the unplanned/early removal of the catheter (e.g., phlebitis or PIVIE) and consider whether this might be a source of pain and discomfort during removal. Consider whether an assistant (potentially an adequately prepared parent) is required to aid in safe removal, support the infant, and provide comfort measures in accordance with local protocol/guidance.

Evidence from numerous sources suggests that following these general considerations can reduce levels of medical error and avoidable patient harm, reduce costs and complaints, enhance the patient and family experience, and ensure that patients and families receive optimal individualized care that supports better short- and long-term developmental outcomes (8–11, 32–38).

#### Removing cyanoacrylate tissue adhesives from the skin

TAs naturally begin to lose their adhesion 5–7 days following application, as the surface of the skin naturally regenerates and exfoliates. In neonates, the skin undergoes post-birth maturation processes, and reapplication of CSCA around the site it was originally applied to is recommended with any dressing change to ensure ongoing benefits. However, it remains important to avoid overapplication (20).

### Pre-CSCA use precautions

Neonates are at risk from numerous iatrogenic complications from VA procedures. Utilizing mnemonics, such as the “5Rs for VA” or “PIV5rights” (27, 39), before insertion can reduce harm. In addition, formal skin condition scoring using a validated tool, e.g., the Braden QD or Neonatal Skin Condition Score (NSCS) (40–42), can be useful for identifying risks and aiding objective assessment and documentation.

Preventing MARSI begins before the application of medical adhesive. Preventative strategies include appropriate adhesive selection for the intended purpose and the application of non-alcoholic silicone skin barrier preparations**.** Evidence suggests that the preapplication of skin barrier products (e.g., Cavilon^TM^ No Sting Skin Barrier Film, 3M Saint Paul, MN, USA.) protects the skin from MARSI and epidermal stripping. It should be noted that while authoritative guidance supports their use (e.g., 42, 43) most skin barrier product use is “off-label” in neonates.

### Removing CSCA

Generally, in neonates, gentle rolling manipulation of the CSCA plaque in a horizontal direction avoiding vertical lifting is all that is required to aid the removal of the adhesive and catheter. Unless it is essential for ongoing care and management of the VA catheter, it is not necessary to remove all the CSCA residue. It is sufficient to remove enough to facilitate the safe removal of the catheter and any remaining CSCA will naturally separate in time; this reduces the risk of MARSI (16–18, 21).

CSCA can be removed before it naturally separates by applying adhesive removers (20). However, formulations containing acetone, alcohol, or latex can be harmful for neonates or can damage some types of catheters [alcohol softens polyurethane (PUR)]. In general, adhesive removers containing alcohol or acetone are best avoided in neonates. This is to protect against further skin damage and avoid possible toxicities from the absorption of ingredients through the skin (16). Numerous branded skin-compatible formulations, such as Acetone Free Adhesive Remover Pads (Medline Industries Northfield lL, USA), or silicone-based adhesive removers, such as Adapt™ Medical Adhesive Remover (Hollister Incorporated, Libertyville IL, USA) and Esenta™ Adhesive Remover and Sensicare™ Sting-Free Adhesive Releaser (both, Convatec Incorporated Bridgewater NJ, USA), are available and when used with care can facilitate easier CSCA removal in difficult cases. If adhesive removers are used then interventions to limit exposure, such as avoiding contaminating adjacent skin and pooling underneath the infant and promptly removing any residue, should be implemented.

### Documentation

Adhere to the principles of proper documentation practices and institution requirements. This will ensure compliance with the expectations of professional practice and that the care provided to the patient and family is accurately recorded (33). The documentation includes the reason for catheter removal, date and time of removal, skin and catheter site condition assessment and any recommendations for ongoing care and observation, effectiveness of comfort interventions, and education to the parents. Document adverse outcomes using incident reporting as per institution guidelines.

## Conclusions

Neonatal VA is a complex undertaking, one in which continual balancing and rebalancing of relative risks and benefits is required to optimize therapy and minimize harm. Opening discussion and debate about VA practice is essential for improving patient outcomes. The motivation for this article emerged from a concern that in reducing one aspect of risk, i.e., catheter dislodgment from better catheter securement, we mitigate risk elsewhere.

This article summarizes the key points (Box 1) for safe CSCA removal and contributes to knowledge in several ways. First, it provokes discussion around an increasingly common feature of everyday practice (the use of CSCA with VA) by highlighting the current state of evidence supporting practice. Second, it draws attention to some of the significant gaps in the understanding of the risks associated with CSCA use with neonatal patients.

BOX 1 **Key points for ensuring safe and effective CSCA removal.**

Applying measures to ensure more effective CVC tip placement at the first attempt will reduce the need for CVC tip repositioning and CSCA removal.

Applying measures to reduce modifiable factors affecting PIVC placement will reduce the incidence of infusion therapy-related complications and need to remove the PIVC.

Avoiding overapplication of the CSCA during catheter securement will enable a better bond and easier removal.

Avoid using alcohol-, acetone-, or latex-containing adhesive removers.

Gentle rolling manipulation of the CSCA plaque is an effective way of removing CSCA residue and facilitating catheter removal.

Awaiting CSCA debonding through natural skin exfoliation is the safest and most effective way of removing TA residues from the skin.

## Data Availability

The original contributions presented in the study are included in the article/Supplementary Material, further inquiries can be directed to the corresponding author/s.
